# miR-148a regulation interferes in inflammatory cytokine and parasitic load in canine leishmaniasis

**DOI:** 10.1371/journal.pntd.0011039

**Published:** 2023-01-31

**Authors:** Gabriela Torres Rebech, Jaqueline Poleto Bragato, Sidnei Ferro Costa, Jéssica Henrique de Freitas, Marilene Oliveira dos Santos, Matheus Fujimura Soares, Flávia de Rezende Eugênio, Paulo Sérgio Patto dos Santos, Valéria Marçal Felix de Lima

**Affiliations:** Department of Clinical Medicine, Surgery and Animal Reproduction, São Paulo State University (UNESP), School of Veterinary Medicine, Araçatuba, Brazil; University of Texas at El Paso, UNITED STATES

## Abstract

Canine leishmaniasis (CanL) is a severe public health threat. Infected animals mediate transmission of the *Leishmania* protozoan to humans via the sandfly’s bite during a blood meal. CanL progression depends on the degree of suppression of the immune response, possibly associated with microRNAs (miR), which can modulate mRNA translation into proteins and (consequently) regulate cell function. Increased miR-148a in splenic leukocytes (SL) of dogs with CanL was observed in previous studies, and in silico analysis, identified possible pathways involved in immune response regulation that are affected by this miR. Therefore, we evaluated the involvement of miR-148a in the regulation of TNF-α, IL-6, IL-12, IL-1β, iNOS, MHCII, CD80, CD3, T-bet, and GATA-3 transcription factors and their relationship with parasite load in SL of dogs with CanL. Splenic leukocytes obtained from healthy and diseased dogs were transfected with miR-148a mimic and inhibitor oligonucleotides. After 48 hours, expression levels of MHCII, CD80, iNOS, CD3, T-bet, and GATA-3 were evaluated by flow cytometry, and concentrations of TNF-α, IL-12, IL-6, and IL-1β were measured in culture supernatants by capture enzyme-linked immunosorbent assays. Transfection of SL with miR-148a mimics decreased iNOS levels in cells and TNF-α, IL-6, and IL-12 in the supernatants of cultured SL from CanL dogs. Interestingly, transfection with miR-148a inhibitor decreased parasite load in SL cells. These results suggest a direct or not regulatory role of this miR in the immune response to *Leishmania infantum* infection. We conclude that miR-148a can modulate immune responses by regulating inflammatory cytokines during CanL. Our results contribute to understanding the complex host/parasite interaction in CanL and could assist the development of treatments.

## Author summary

The *Leishmania infantum* is a protozoan parasite that causes visceral leishmaniasis (VL), the most lethal form of the disease in humans. In the domestic environment, dogs are the primary reservoir of this parasite; the infection may present as slimming, extensive skin lesions, excessive nail growth, and others. Treatments for canine leishmaniasis (CanL) are ineffective because treated dogs often re-develop signs and symptoms when the medication is stopped. However, the immune response determines the presentation and cellular immune responses are most effective for parasite replication control. Resistance depends on the inflammatory or microbicidal activity of cytokines. Nevertheless, the suppression of this immune response is evident during CanL and is related to many factors. Recent reports of dogs with CanL found increased small RNAs called microRNAs (miR) that regulate cell function and might be associated with the immune regulation observed during this disease. Therefore, we inhibited and increased levels of a specific miR (miR-148a) *in vitro* in cells of dogs (*Leishmania*-infected/sick and uninfected/heathy) and measured immunological markers. We observed a reduction of some essential inflammatory cytokines when the miR was increased and parasite number reduction when the miR was inhibited. These results suggest that this miR influences immunological responses in dogs with CanL related to disease progression. The findings contribute to the understanding of immunological aspects of CanL, facilitating the development of immunotherapeutic approaches.

## Introduction

Visceral leishmaniasis (VL) is a neglected disease caused by the protozoan *Leishmania*; it is transmitted by the bite of infected female phlebotomine sandflies [1]. Visceral leishmaniasis is the most severe form of the disease [1,2]; in 2019, Brazil reported 97% of all VL cases in the Americas [1]. Domestic dogs are natural reservoirs of *Leishmania* in urban areas [2,3] because they present a high parasite load in the skin and live near humans, facilitating transmission [4–6].

During CanL, the spleen is among the most parasitized [7–9] and where the major immune response in leishmaniasis occur [10]. Spleens of infected animals display a mixed Th1/Th2 immune response [8]; symptomatic dogs show predominantly Th2 responses that contribute to parasite development [11,12]. In contrast, resistance to CanL has been associated with IL-12-induced Th1 protective immune responses and pro-inflammatory cytokine production [13,14] (IFNγ, IL-12, IL-6, TNF [15]), inducible nitric oxide synthase (iNOS) [16] and nitric oxide (NO) [17] leishmanicidal activity.

*Leishmania* infection can suppress host immune responses [18]. High parasite load implies low cytokine expression in the canine spleen [15]. Low splenic iNOS [16] and IL-12 mRNA [15] levels have been associated with parasite persistence during CanL. Nevertheless, TNF-α is increased in the spleen during CanL, and this cytokine was related to disease process evolution [9].

MicroRNAs (miR) are small non-coding RNAs that mediate post-transcriptional gene regulation [19,20], affecting immune response in inflammatory diseases [20]. Recent studies demonstrated changes in miR expression in peripheral blood mononuclear cells [21] and splenic leukocytes (SL) [22] from dogs naturally infected with *L*. *infantum*.

Microarray analysis revealed a 2.29-fold increase of miR-148a in SL during CanL. *In silico* analysis revealed canonical pathways directly regulated by miR-148a. These included PTEN signaling, cytotoxic T lymphocyte-mediated apoptosis of target cells, the Th1 and Th2 activation pathways, and antigen presentation, among other pathways related to cytokine production regulated by this miR [22].

MicroRNA-148a is a member of the miR-148/-152 family located on human chromosomes 7, 12, and 17, mouse chromosomes 6, 15, and 11 [23]. This miR can modulate innate and adaptive immunity genes, particularly antigens of the major histocompatibility complex (MHC) [23,24]. It is also involved in cancer initiation, tumor growth, and metastasis [23].

According to the miRBase Sequence Database (a database of published miRNA sequences), cfa-miR-148a is encoded in the cromossome 14 and presents complete homology between mice and humans [25]. Studies showed that miR-148a reduced IL-6, IL-12, IL-1β, and TNF-α in experimentally induced inflammatory disease in a human microglial cell line [26] and murine models [24,27]; however, the role of miR148 in immune regulation of CanL has not yet been studied.

In the present study, we demonstrated that transfection with a miR-148a mimic resulted in downregulation of pro-inflammatory cytokines (iNOS, TNF-α, IL-6, and IL-12), and a miR-148a inhibitor reduced parasitic load in cultured SL obtained from dogs naturally infected with *L*. *infantum*.

## Methods

### Ethics statement

The Animal Experimental Research Ethics Committee approved the study, and we received the approval of the Animal Use Ethics Committee of UNESP–Universidade Estadual Paulista "Júlio de Mesquita Filho” (process 00624–2018).

### Animal screening and sample collection

The control group consisted of five healthy mongrel animals 2–5 years old (three females and two males) without hematological and biochemical alterations, that presented no detectable anti-*Leishmania* antibodies according to immunochromatographic (Dual-Path Platform DPP) and enzyme-linked immunosorbent assay (ELISA) [28], and no detection of *Leishmania* DNA by molecular analyses (S1 Table) [29].

The CanL group consisted of fourteen mongrel dogs (2–5 years old) naturally infected with *L*. *infantum*. These dogs came from the Araçatuba Zoonoses Control Center and presented detectable anti-*Leishmania* antibodies according to immunochromatographic (Dual-Path Platform DPP) and enzyme-linked immunosorbent assay (ELISA) [28], and detection of *Leishmania* DNA by molecular analyses [29]. All dogs were symptomatic and had at least three clinical signs: onychogryphosis, weight loss, ear-tip injuries, lymphadenomegaly, cachexia, alopecia, or periocular and skin lesions (S1 Table).

Blood samples were obtained from the jugular vein puncture from both groups and placed in tubes without anticoagulant to obtain serum for indirect ELISA [25] (S1 Table) and biochemical profile (S2 Table) and tubes containing EDTA (BD, USA) to perform complete blood counts (S3 and S4 Tables). All samples were processed immediately after collection.

Infected dogs were euthanized using thiopental (Cristália Itapira, SP, Brazil), followed by intravenous 19.1% potassium chloride injection by the same route, as recommended for VL control and in situ compliance with local legislation. After euthanasia, 2 cm^3^ spleen fragments were collected for SL isolation. Spleen fragments in control dogs were removed by surgical excision [12]. All spleen fragments were maintained in complete RPMI-1640 medium (Sigma, USA) supplemented with 10% heat-inactivated fetal bovine serum (Gibco, USA), 0.03% L-glutamine (Sigma, USA), 100 IU/mL of penicillin (Sigma, USA), and 100 mg/mL of streptomycin (Sigma, USA), for SL isolation until processing.

### Splenic leukocyte isolation

Splenic leukocytes were obtained from 2-cm^3^ fragments that were macerated and added to 10 ml of RPMI-1640 medium (Sigma, USA) supplemented with 10% heat-inactivated fetal bovine serum, 0.03% L-glutamine, 100 IU/mL penicillin, and 100 mg/mL streptomycin. After removing cell debris with a 100-μm cell strainer (BD Falcon Cell strainer, USA), suspensions were processed with 5 mL of red blood cell lysis buffer containing 8 g/L ammonium chloride 1.6 g/L EDTA, and 0.16 g/L calcium carbonate at 4°C for 10 minutes. This material was centrifuged at 2000 rpm for 5 minutes and washed with phosphate-buffered saline (PBS) at pH 7.2 three times. Cells were then counted in a Neubauer chamber.

### Serological diagnosis by ELISA

Serum samples were analyzed by ELISA assay using total antigen from lysed promastigotes [28]. The antigen was coated overnight with 20 μg/ml protein (pH 9.6), then washed three times in PBS containing 0.05% Tween 20 (washing buffer) and saturated for 1 hour with 150 μl/well of a mixture of PBS and 10% FBS at room temperature. Next, the preparation was rewashed three times with washing buffer. Blocking buffer/Tween (100 μl of serum sample (1/400) diluted in PBS, pH 7.2, containing 0.05% Tween 20 and 10% FCS) was added to each well and incubated at room temperature for 3 h, followed by three washes with washing buffer. Subsequently, 100 μl/well of anti-dog IgG conjugated with horseradish peroxidase (Sigma, St. Louis, MO, USA) at appropriate dilution in blocking buffer/Tween was added, incubated at room temperature for 1 hour, and washed. Substrate solution (0.4 mg/ml o-phenylenediamine (Sigma) and 0.4 μl/ml hydrogen peroxide in phosphate citrate buffer, pH 5.0) was added at 100 μl/well and developed for 5 min at room temperature. The reaction was stopped with 50 μl of 3M sulfuric acid. Absorbance was measured at 490 nm using a Tecan microplate reader (Sunrise model ref. 16039400).

Negative and positive controls were included in each plate. Positive controls obtained from a hyperimmune animal were included. The cutoff was determined using the mean +3 standard deviations of the readings obtained from post-transfection, performed using the commercial mirVana kit for isolation serum samples of healthy dogs from areas non-endemic for leishmaniasis.

### DNA extraction and determination of *Leishmania* species

Extraction of total DNA from 5 x 10^4^ SL with a commercial kit (Kit Dneasy [Qiagen]) was performed following the manufacturer’s instructions. Final DNA elution was 50 μL. Determination of the *Leishmania* species was performed using restriction fragment length polymorphism-polymerase chain reaction (RFLP-PCR) [29], comparing the restriction profiles of the sample with profiles obtained from *L*. *infantum* (IOC / L0575-MHOM / BR / 2002 / LPC-RPV), *L*. *braziliensis* (IOC / L0566-MHOM / BR / 1975 / M2903) and *L*. *amazonensis* (IOC / L0575-MHOM / BR / 1967/ PH8) as positive controls, and water as a negative control (S1 Fig).

### Extraction and quantification of total RNA

Extraction of total RNA, including miRNAs, from 5 x 10^4^ SL was performed using a mirVana Kit with phenol (Invitrogen), following the manufacturer’s instructions. After RNA isolation, samples were stored at –80°C. RNA samples were analyzed using a spectrophotometer (NanoDrop, Thermo Scientific, USA) for purity evaluation (260 nm/280 nm) and quantification.

### Real-time PCR for miR-148a

To confirm the upregulation of miR-148a in dogs with CanL [22], real-time PCR (qPCR) was performed. cDNA production was performed using the miScript RT II kit (Qiagen, USA), as recommended by the manufacturer. A total of 1 μg of RNA was used for each sample with the 5x miScript Hiflex Buffer in a final volume of 20 μl. The mix was incubated for 60 min at 37°C, followed by 5 min at 95°C to inactivate the miScript reverse transcriptase. Next, qPCR was performed using a commercially available specific primer for the *Canis familiaris* miR-148a and the endogenous reference RNA SNORD96A (miScript, Qiagen) [21,22]. The SYBR Green system (miScript SYBR Green PCR Kit, Qiagen) was used in a real-time thermal cycler (Mastercycler-Ep realplex-4S, Eppendorf). Amplification conditions consisted of an initial activation step of 95°C for 15 min followed by 40 cycles of 94°C for 15 seconds, 55°C for 30 seconds, and 70°C for 30 seconds (for denaturation, annealing, and extension, respectively). A standard curve was generated for miRNA analysis using a serial dilution of a pool of cDNAs (maximum and minimum concentration of 0.163 ng/μL and 0.25468x10^-2^ ng, consecutively, measured using a Qubit dsDNA HS Assay Kit following manufacture instructions in spectrophotometer (Qubit, Thermo Scientific, USA) and obtained a linear amplification of 0.955 (S5 Table). Relative gene expression of miR-148a was performed by converting the sample cycle threshold values to a concentration (ng/μl) based on the standard curves and we divided the mean of this values by means values of SNORD96A. Individual CT values of miR-148a, SNORD96A and relative miR-148a expression which of dogs are describe in S6 Table. All samples were run in duplicate. Meltin curve showed single peaks for each target (S2 Fig).

### Transfection with miR-148a mimic and inhibitor in SL

Splenic leukocytes were cultured (1.6 x 10^5^ cells/replicate) in 24-well plates in triplicate, for 48 h at 37°C in 5% CO_2_. Reagents Scrambled (All Stars Negative control siRNA Qiagen, USA) (5 nM), miR-148a mimic (5 nM), and miR-148a inhibitor (50 nM) (miScript miRNA mimic and inhibitor Qiagen, USA) was used, and SL was transfected using 3 μL of Hiperfect (Qiagen, USA) in each well, following manufacturer’s instructions.

To evaluate transfection rates, Cell Death Control (AllStars HS siRNA Qiagen, USA) was used at a final concentration of 50 nM. According to the manufacturer’s instructions, the fluorescence rate was measured using flow cytometry with 7-AAD Viability Staining Solution (BioLegend, USA). Cell death was measured using trypan blue in a Neubauer chamber and optical microscopy. A medium transfection rate of 20% was obtained for both groups.

### Flow cytometry analysis of SL

For flow cytometry analysis, 1 x 10^4^ of SL were incubated with Fc blocking buffer (10% of FBS) for 30 min at room temperature. Splenic leukocytes was centrifuged at 1800 rpm for 7 minutes and then incubated with anti-dog MHC class II conjugated with fluorescein isothiocyanate (FITC) (Bio-Rad, USA) or mouse anti-dog CD3/FITC (Bio-Rad) following manufacturer’s instructions. The SL incubated with MHC class II were also marked with anti-mouse CD80 monoclonal antibody conjugated to phycoerythrin (PE) (Biolegend) or rabbit anti-mouse iNOS polyclonal/PE (Byorbit) following manufacturer’s instructions. Splenic leukocytes incubated with mouse anti-dog CD3/FITC were marked with mouse anti-human GATA-3 monoclonal/PE (Biolegend) or mouse anti-human T-bet monoclonal/PE (Biolegend) following manufacturer’s instructions. Finally, anti-MHCII/CD80, MHCII/iNOS, CD3/T-bet, and CD3/GATA-3 double-tagged SL were analyzed.

Anti-CD80 and anti-iNOS antibodies possess predicted reactivity described in datasheets. Anti-GATA-3 and anti-T-bet antibody are between humans (GenBank, accession # NP_037483 and CAA38877) and dogs (XP_548164 and XP_005617214, 93% and 96%, respectively). To avoid non-specific binding, cells were incubated with their respective control isotypes.

For parasite load quantification, we used a previously described method [30] with modifications. After 48 h in culture, cells were harvested and incubated for 60 minutes at room temperature in 1x PBS containing 1% paraformaldehyde (Sigma). Then, they were centrifuged at 400 *g* for 5 minutes, and permeabilization of the membrane and the parasitophorous vacuole was performed by resuspending the cells in ethanol (Sigma) for 60 minutes at –20°C. Cells were washed with 2% BSA in PBS 1x and incubated for 60 minutes at 4°C with anti-LPG-Leishmania monoclonal antibody (ABD, Serotec) diluted 1:250. After three consecutive washes with PBS/BSA, amastigotes were stained with 1 μM of anti-IgG2a conjugated to PE (Sigma) and with monoclonal antibody anti-human CD14 conjugated to FITC (Bio-Rad) for 60 min at 4°C. Cells were washed in PBS/BSA buffer and stored at 4°C in the dark until analysis. All acquisitions were counted in 10,000 events by experimental replicates on channels FL1 and FL2, and cytometric analysis was performed using an Accuri C5 Flow Cytometer (BD Biosciences, USA) with BD Accuri C6 software, version 1.0.264.21 (BD Biosciences, USA).

### Capture ELISA for TNF-α, IL-12, IL-6, and IL-1β quantification

After 48 h of transfection, supernatants from the cultured SL were collected, centrifuged at 2500 rpm, and stored at –80°C until analysis. According to the manufacturer’s instructions, cytokine measurements were performed using DuoSet ELISA Development Systems kits (R&D Systems, Minneapolis, MN, USA).

### Statistical analysis

Data were subjected to normality tests (D’Agostino & Pearson omnibus, Shapiro-Wilk, and Kolmogorov–Smirnov), and non-parametric tests were used. The miR148, CD80, CD3, GATA-3, MHCII, iNOS, and T-bet expressions were compared in SL between control and infected groups using the Mann-Whitney test. After transfection of SL with mimics and inhibitors of miR-148a, CD80, CD3, GATA-3, MHCII, iNOS, and T-bet expression were evaluated using the Friedman test with Dunn multiple comparisons. Differences were considered significant when p < 0.05. GraphPad Prism 6 (Graph Pad Software, Inc. CA. USA) was used to perform data analysis.

## Results

### All CanL dogs were infected by *Leishmania infatum*

First, we performed determination of *Leishmania* species by RFLP-PCR [29] to verify which specie the dogs were infected, and we observed that all dogs of CanL group were infected by *Leishmania infantum* (S1 Fig).

### miR-148a expression is increased in SL of dogs with CanL

**Fig 1:**
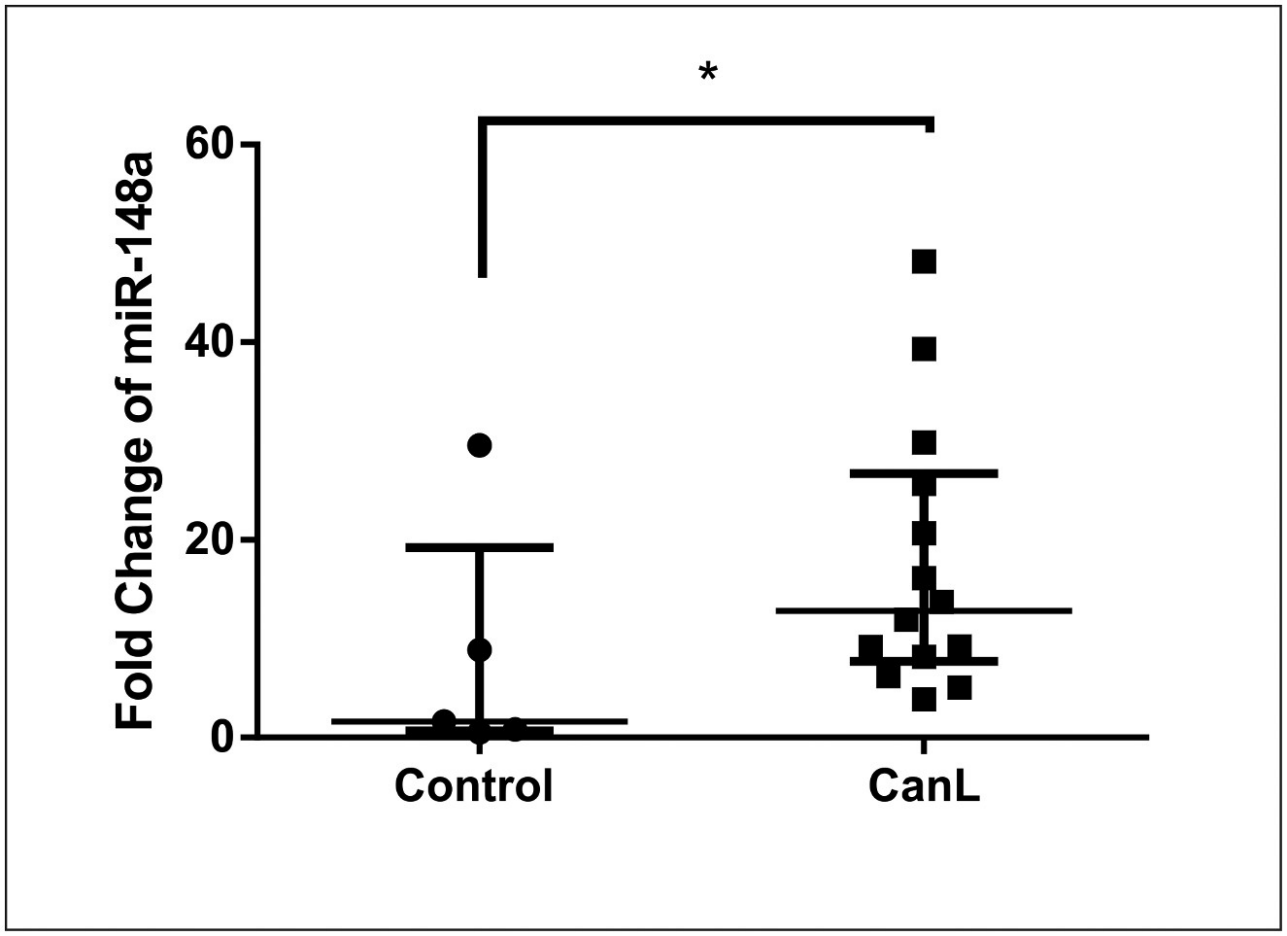
Expression of miR-148a during CanL. miR-148a expression was quantified using real-time PCR in SL of control and CanL groups. Bars represent miR-148a expression median values (1.616 and 12.82), 25^th^ (0.6716 and 7.701) and 75^th^ (19.21 and 26.71) percentile interquartile range from control and CanL groups, respectively. Symbols represent data from individual animals. Asterisks represent significant differences (Mann-Whitney test, p = 0.03).

To confirm the increase in miR-148a expression in CanL, real-time PCR was performed with SL from dogs in both groups. We observed a higher expression of miR-148a in the CanL group (median values 12.82) than in the control group (median values 1.61) (Fig 1).

### Canine leishmaniasis regulates CD80, CD3, and GATA-3 expression in cultured SL

**Fig 2:**
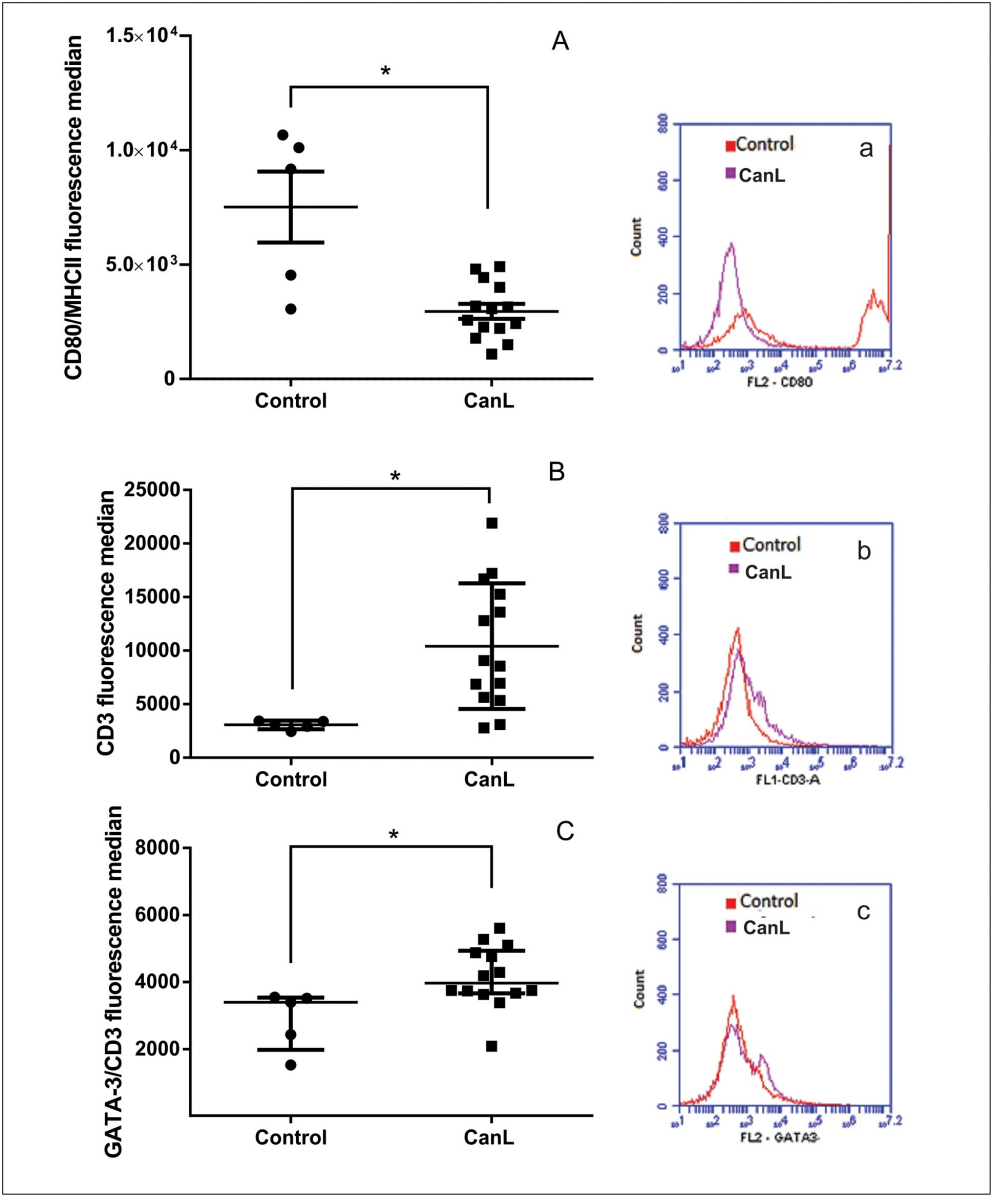
Expression of CD80/MHCII, CD3 and GATA-3/CD3 in SL culture. Expression of CD80 in MHCII^+^ (CD80/MHCII) leukocytes (A), CD3 (B) and GATA-3 in TCD3^+^ leukocytes (GATA-3/CD3) (C). Splenic leukocytes from control (healthy dogs) and CanL (dogs naturally infected by *L*. *infantum)* groups, were maintained in culture for 48 h at 37°C and 5% CO_2_ without any treatment and, posteriorly, were marked with monoclonal antibodies conjugated to fluorochromes for analyze by flow cytometry. Bars represent expression median values of CD80/MHCII (9177 and 2818), CD3 (3144 and 8816) and GATA-3/CD3 (3397 and 3971), 25^th^ percentile interquartile range of CD80/MHCII (3802 and 2103), CD3 (2684 and 5565) and GATA-3/CD3 (1979 and 3661) and 75^th^ percentile interquartile range of CD80/MHCII (10,389 and 4,109), CD3 (3406 and 15616) and GATA-3/CD3 (3533 and 4931) from control and CanL groups, respectively. Symbols represent data from individual animals. Asterisks represent significant differences (Mann-Whitney test, p = 0.01 (A), p = 0.007 (B), p = 0.007 (C)). Representative histogram of overlay protein expression analysis showed significant differences: CD80/MHC (a), CD3 (b), and GATA-3/CD3 (c) between control (red) and infected (purple) groups.

Considering immune response regulation during CanL [18], expression of MHCII, CD80 in MHCII^+^ leukocytes, iNOS in MHCII^+^ leukocytes, CD3 and transcription factor T-bet in TCD3^+^ leukocytes and GATA-3 in TCD3^+^ leukocytes, was analyzed after SL culture from control and CanL groups without any treatment. We observed that the proportions of SL MHC^+^ or TCD3^+^ of total analyzed cells (10,000 cells), were 19.77% and 6% in the control group, 38.88% and 11.03% in the infected group, respectively. Expression of CD80 protein was lower in MHCII^+^ cells in the CanL group (means values 2818) when compared with control group (means values 9177) (Fig 2A), whereas expression of CD3 (Fig 2B) and GATA-3 in TCD3+ lymphocytes (Fig 2C) was higher in the CanL group than the control group (means values of CD3 (3144 and 8816) and GATA-3 (5565 and 2684) from CanL and control group respectively). Expression of MHCII and iNOS in MHCII^+^ leukocytes and transcription factor T-bet in TCD3+ lymphocytes showed no significant differences between groups (S3 Fig). Fig 2 displays a representative overlay histogram of proteins expression analysis showing significant differences in CD80/MHC (2a), CD3 (2b), and GATA-3/CD3 (2c) between control (red) and infected (purple) groups.

### miR-148a mimic transfection decreases iNOS expression in CanL

**Fig 3:**
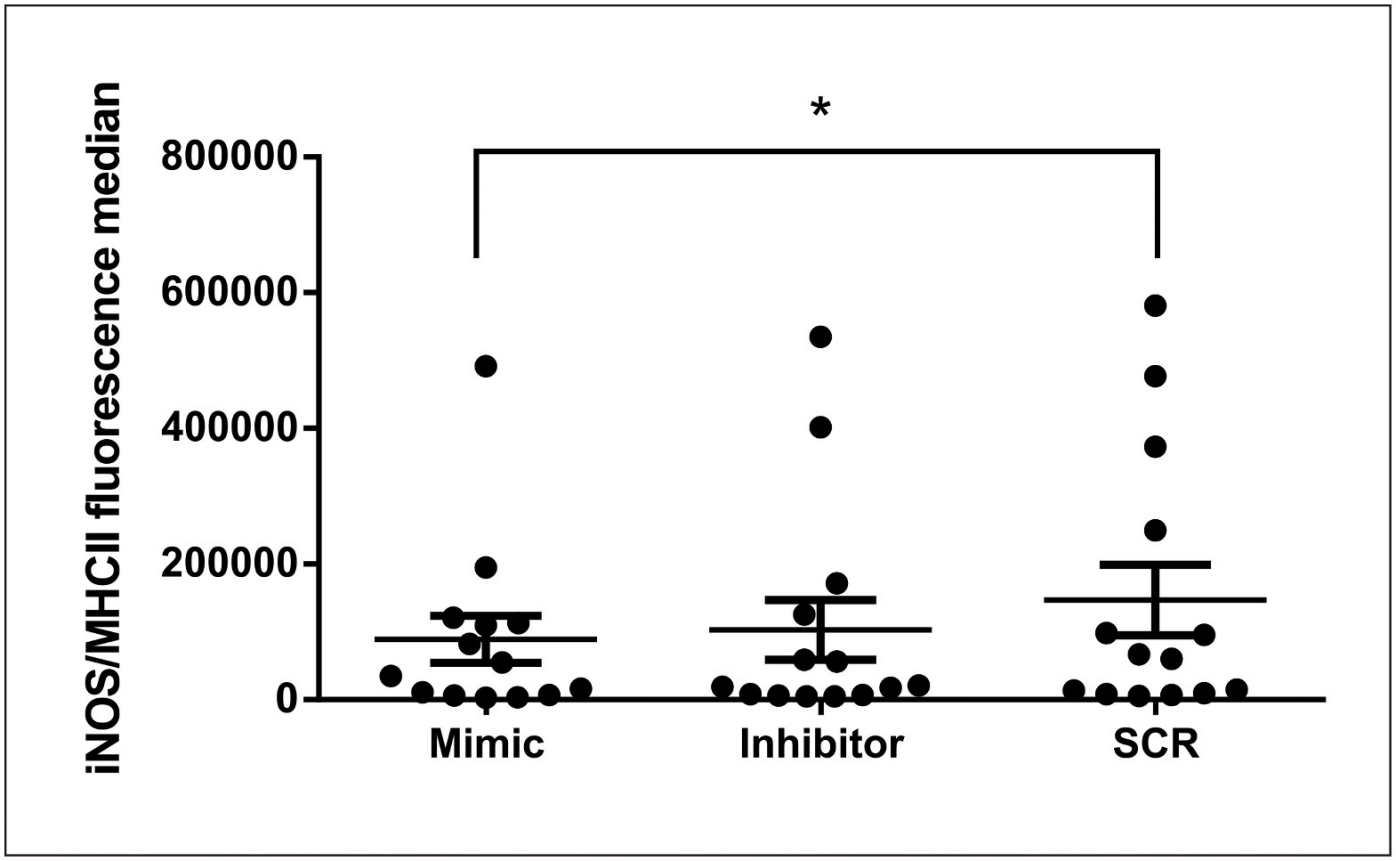
Expression of iNOS in MHCII^+^ leukocyte with mimic or inhibitor of miR-148a. iNOS expression was evaluated in SL from CanL group (dogs naturally infected by *L*. *infantum*) transfected with miR-148a mimic or inhibitor, or SCR and maintained in culture for 48 h at 37°C and 5% CO_2_. Posteriorly, SL were marked with monoclonal antibodies conjugated to fluorochromes for analyze by flow cytometry. Bars represent iNOS expression median values (45099, 20237 and 63677), 25th (7064, 6716 and 9194) and 75^th^ (115028, 136904 and 280471) percentile interquartile range of mimic, inhibitor and SCR treatments, respectively. Symbols represent data from individual animals. Asterisks represent significant differences (Friedman test with Dunn’s multiple comparison test, p = 0.008).

Following confirmation that miR-148a was increased in SL from dog with CanL, we investigated the role of this miR through transfection with a mimic and an inhibitor of miR-148a. Transfected SL were cultured at 37°C and 5% CO_2_ for 48 h, when the expression of proteins, MHCII, CD80, iNOS, CD3, T-bet, and GATA-3 was evaluated using flow cytometry. We did not find significant differences in the control group following treatment with mimic or inhibitor of miR-148a (S4 Fig). In contrast, in the CanL group, miR-148a mimic reduced iNOS in MHCII^+^ leukocytes compared to scrambled (SCR) (Fig 3). Expression of MHCII, CD80 in MHCII^+^ leukocytes, CD3, T-bet in TCD3^+^ leukocytes, and GATA-3 in TCD3^+^ leukocytes did not vary between treatments in the infected group (S5 Fig).

### Canine leishmaniasis regulates TNF-α and IL-1β expression in supernatants of cultured SL

**Fig 4:**
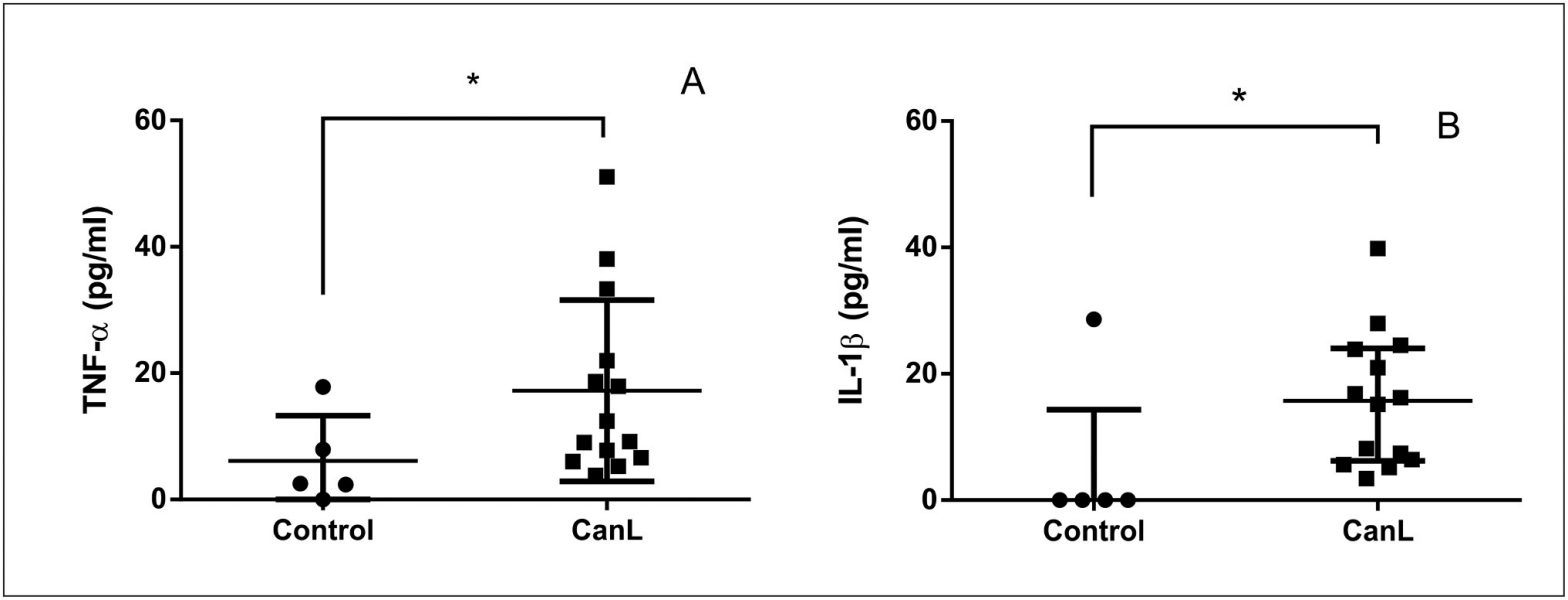
Concentration of TNF-α and IL-1β during CanL. TNF-α (A) and IL-1β (B) were quantified, by capture ELISA, in SL supernatants cultures from control (dogs without *L*. *infantum* infection) and CanL (dogs naturally infected by *L*. *infantum)* groups. Splenic leukocytes were maintained in culture for 48 h at 37°C and 5% CO_2_, without treatment. Bars represent concentration median values of TNF-α (2.51 and 10.81) and IL-1β (0.0 and 15.70), 25th percentile interquartile range of TNF-α (1.18 and 6.49) and IL-1β (0.0 and 6.238) and 75th percentile interquartile range of TNF-α (12.89 and 24.80) and IL-1β (0.0 and 15.70) from control and CanL groups, respectively. Symbols represent data from individual animals. Asterisks represent significant differences (Mann-Whitney test, p = 0.04 (A and B).

We compared TNF-α, IL-6, IL-12, and IL-1β levels in cultured SL supernatants from dogs of control and CanL groups to investigate immune response modulation during CanL. We observed that dogs with CanL presented increased levels of TNF-α (Fig 4A) and IL-1β (Fig 4B), whereas IL-6 and IL-12 did not differ between groups (S6 Fig).

### miR-148a mimic transfection decreases TNF-α, IL-6, and IL-12 concentration during CanL

**Fig 5:**
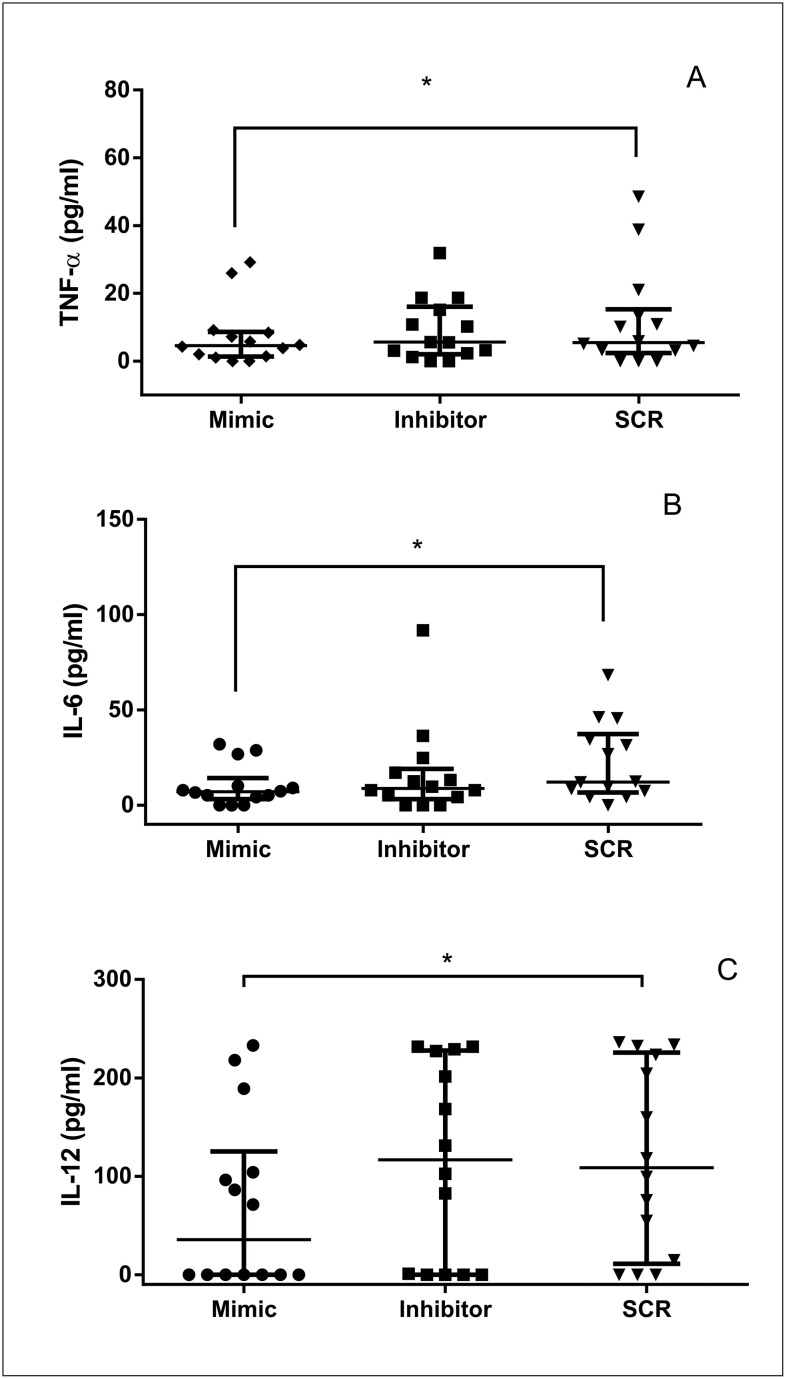
Concentration of TNF-α, IL-6, and IL-12 after transfection in SL with miR-148a mimic or inhibtor. TNF-α (A), IL-6 (B), and IL-12 (C) were quantified in SL supernatants cultures from CanL group (dogs naturally infected by *L*. *infantum*) and transfected with miR-148a mimic or inhibitor, or SCR in the presence of Hiperfect following 48 h in the culture at 37°C and 5% CO2. Bars represent concentration median values of TNF-α (4,570, 5,609 and 5,490), IL-6 (7,072, 8,885 and 12,25) and IL-12 (35,66, 116,8 and 108,7), 25th percentile interquartile range of TNF-α (1,332, 2,065 and 2,376), IL-6 (3,273, 3,273 and 6,706) and IL-12 (0,0, 0,0 and 11,07) and 75th percentile interquartile range of TNF-α (8,628, 16,06 and 15,30), IL-6 (14,36, 19,10 and 37,42) and IL-12 (125,2, 227,7 and 225,7) of mimic, inhibitor and SCR treatments, respectively. Symbols represent data from individual animals. Asterisks represent significant differences (Friedman with Dunn’s multiple comparison test, p = 0.03 (A), p = 0.01 (B), p = 0.007 (C)).

To determine whether miR-148a regulates TNF-α, IL-6, IL-12, and IL-1β production, we compared these cytokines in SL culture supernatants from control and CanL groups after transfection with miR-148a mimic or inhibitor. We did not find significant differences in the control group regardless of treatment (S7 Fig). However, in the CanL group, TNF-α (Fig 5A), IL-6 (Fig 5B), and IL-12 (Fig 5C) concentrations were lower in the presence of miR-148a mimic than in the SCR transfection. IL-1β production did not differ significantly between transfection conditions in the CanL group (S8 Fig).

### Inhibition of miR-148a reduces parasitic load in SL culture from dogs with CanL

**Fig 6:**
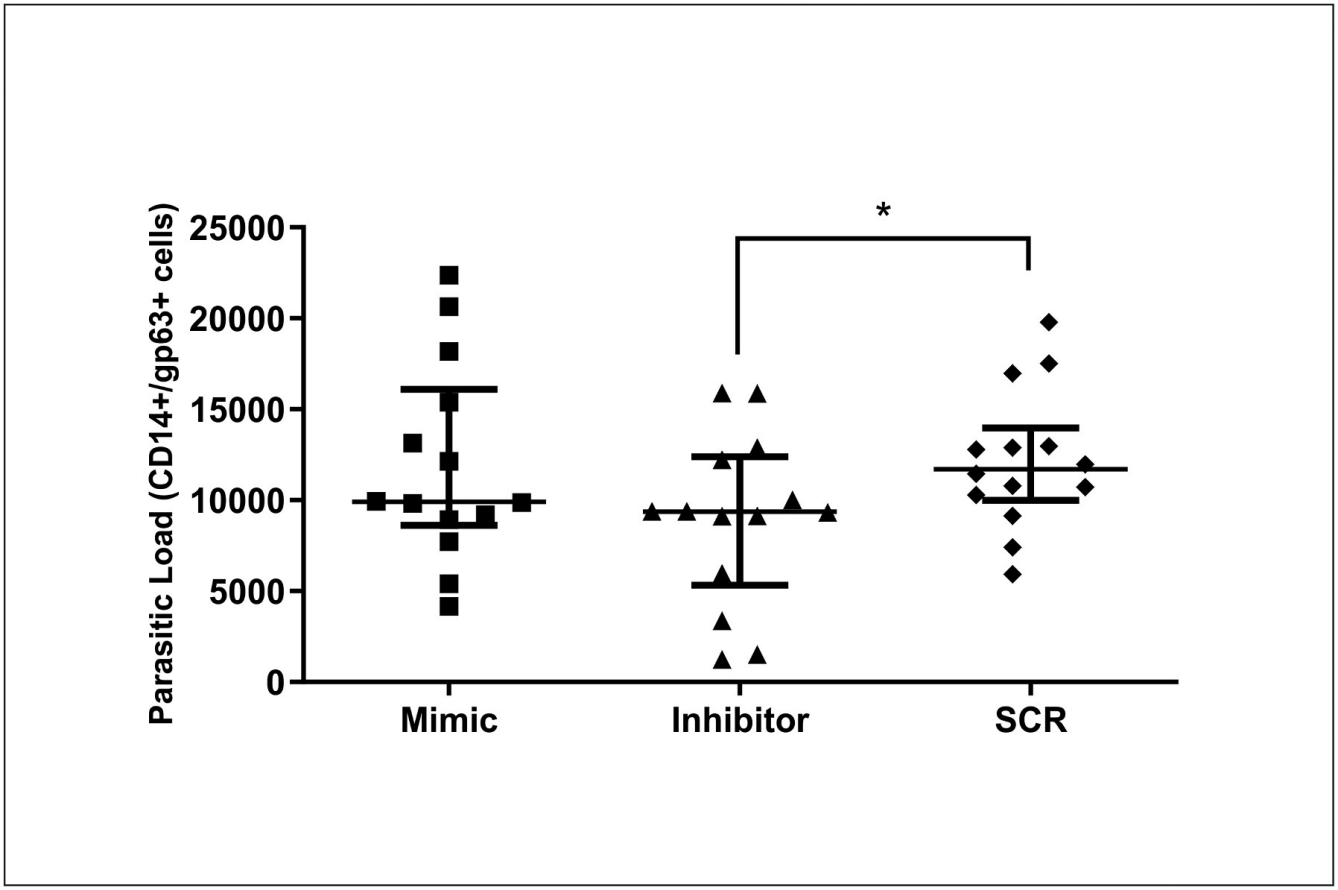
Parasitic load of SL. The parasitic load was quantified in splenic leukocyte cultures of dogs naturally infected by *L*. *infantum* and transfected with miR-148a mimic, miR-148a inhibitor, and SCR transfection control in the presence of Hiperfect following 48 h in the culture at 37°C and 5% CO_2_. Bars represent parasitic load median values (9904, 9357 and 11707), 25th (8623, 5323 and 9992) and 75^th^ (16086, 12380 and 13975) percentile interquartile range of mimic, inhibitor and SCR treatments respectively. Symbols represent data from individual animals. Asterisks represent significant differences (Friedman with Dunn’s multiple comparison test, p = 0.02).

To determine whether miR-148a plays a role in regulating *L*. *infantum* infection, we measured parasitic load using flow cytometry in SL transfected with miR-148a mimic and inhibitor. We found that inhibition of miR-148a reduced parasitic load (Fig 6).

## Discussion

Immune responses observed in naturally infected dogs with symptomatic *L*. *infantum* are characterized by effective cellular response suppression, which may be related to an increase in miRs such as miR-148a. To investigate the role of miR-148a in CanL, we analyzed miR148a targets after transfection of SL from naturally infected dogs by *L*. *infantum* with a mimic and inhibitor of this miR. *In silico* analysis allowed for identifying miR-148a targets, including CD80, iNOS, T-bet, GATA-3, and pro-inflammatory cytokines [22].

We observed reduced expression of CD80 and higher levels of CD3, GATA-3, TNF-α, and IL-1β in dogs with CanL (infected group) than in uninfected dogs (control group). Following transfection, there was increased action of miR-148a (with the help of mimics) that reduced iNOS expression in leukocytes and TNF-α, IL-6, and IL-12 in the supernatants of cultured SL from the infected group. Inhibition of miR-148a reduced parasitic load, suggesting a regulatory role for this miR in immune responses to CanL.

We did not observe changes in MHCII expression in SL between infected cells and control. Similar results were observed in *L*. *infanutm* infected canine macrophages [31]. The MHCII molecule plays a crucial role in adaptive immune response development via antigenic peptide presentation to T lymphocytes [32,33]; however, MHCII expression does not appear to be affected in CanL.

We observed less CD80^+^/MHCII^+^ expression in dogs in the infected group than in the control group. CD80 is a positive co-stimulatory molecule that determines adaptive immune response [18,33], and its absence is involved in T lymphocyte anergy or apoptosis [34] observed during CanL [12]. Another study indicated that CD80 mRNA expression is unaffected in monocyte-derived macrophages from symptomatic dogs with CanL [18]. Thus, these results suggests a development of negative post-transcriptional regulation of CD80. Low expression of this molecule can impair antigen presentation by antigen presentation cells (APC) and result in ineffective immune response resulting in disease progression.

In CanL, iNOS expressed by macrophages in the spleen was negatively associated with parasitic load, suggesting possible deregulation of this molecule in the disease, ensuring the stability of the protozoan in infected cells [16]. In fact, we observed decrease of iNOS expression in SL from the infected group following transfection with mimic suggesting miR-148a participation in regulation of this molecule. Our study is the first to report miR-148a participation in iNOS regulation in CanL, suggesting that this miR interferes with canine immune responses to *Leishmania*, as previously observed with miR-21 [22].

Splenic leukocytes from the infected group showed greater expression of CD3 than the control group. A similar result was observed in other tissues of dogs severely affected by leishmaniasis [35,36]. The observed increase in CD3 during CanL, suggests that there are a proliferation of T cells, however, this cells are not developing a leishmanicidal effective function, with possible downregulation of critical proteins for inducing cell-type immune responses, contributing to disease progression in dogs. Therefore, miR-148a regulation did not change CD3 expression in our work, probably there is no relation of this miR with overexpression of this molecule during CanL.

Although, CD3 was up-expressed in CanL, expression of the transcription factor T-bet in CD3^+^ lymphocytes did not vary between groups in our study, unlike the increase of this transcription factor previously reported in skin [37] and spleen [8] of dogs with CanL. T-bet is related to Th1-type responses and is associated with protozoan [38] and in *L*. *infantum* [39] infection resistance. However, there is an imbalance between Th1/Th2 responses, already evidenced in CanL [11,40–42], which may explain the lack of difference in T-bet expression we observed, reinforcing the idea​ of effective immune response suppression in CanL [37].

On the other hand, we observed increased GATA-3 expression in CD3^+^ lymphocytes from the infected group. GATA-3 mRNA expression is observed in the spleens of dogs in the first month following experimental infection with *L*. *infantum*, with detection of Th2-type cytokine mRNA (i.e., IL-4) [8]. Transcription factor GATA-3 (a precursor of Th2-type responses) is responsible for anti-inflammatory cytokine production that can lead to control of clinical signs, not necessarily accompanied by disease control [37] and is therefore associated with disease progression [41]. Our results reinforce the modulation hypothesis of immune response in CanL [11,40–42]. Increased or decreased miR-148a did not alter T-bet and GATA-3 expression in CD3+ lymphocytes in the infected group, suggesting that miR-148a does not participate in these transcription factors regulation.

The increased TNF-α observed in the infected SL supernatants confirms the involvement of this cytokine, already evidenced in CanL [9] associated with host resistance [37,43]. However, TNF-α decreased in transfected SL culture supernatants after mimic transfection with miR-148a in the infected group, although, is not known if miR-148a has a direct or indirect action. Mouse dendritic cells LPS-stimulated presented similar result [24], confirming the participation of miR-148a in the downregulation of TNF-α. Our study is the first to show the relation of TNF-α expression and miR-148a in CanL.

The disease in dogs did not alter IL-6 levels in SL culture supernatants, as reported in previous studies [44]; however, high IL-6 mRNA expression was found in canine spleens [45]. Taken together, these results suggest post-transcriptional regulation of this cytokine in leishmaniasis, contributing for parasite replication in the host, because, interleukin 6 is a pro-inflammatory cytokine that participates in immune responses, parasite control, and tissue repair [46], therefore, their reduction can be one reason for disease susceptibility. In our study, supernatant concentrations of IL-6 decreased in SL transfected with mimic miR-148a, similar to a previous report in mouse dendritic cells [24], than, these findings suggest that miR-148a is related with IL-6 negatively regulation. We are the first to demonstrate miR-148a regulation interferes in IL-6 in CanL.

Interleukin 12 in SL supernatants did not differ between groups in our study. IL-12 mRNA was absent in *in vitro* infected *L*. *infantum* canine macrophages [18] and in spleens of *L*. *infantum* naturally infected dogs [8]. Interleukin 12 is an essential cytokine for differentiation of Th1 lymphocytes [11]; in dogs with CanL, it was negatively related to parasitic load and was suppressed by the excess of parasites [15,37]. In our study, IL-12 decreased in SL transfected with miR-148a mimic in the infected group. This result suggests that IL-12 mRNA is a target of miR-148a. There are no previous studies reporting the relationship between IL-12 and miR-148a in CanL; however, it has been observed that inhibition of miR21 in CanL leads to restoration of the concentration of this cytokine and reduction in parasitic load [22], suggesting that more than one miR could modulate this cytokine.

In our study, IL-1β increased in the supernatants of SL from naturally infected dogs, confirming previous results and its role in CanL [44]. IL-1β derived from antigen-presenting cells (APCs) is an essential pro-inflammatory cytokine to induce Th1 immune response [47,48]; however, its overabundant production can result in tissue damage [47]. Transfection with miR-148a in SL did not alter IL-1β concentrations in supernatants, suggesting that miR-148a does not contribute for regulation of this cytokine in CanL.

Interestingly, although inhibition of miR-148a did not alter cytokine concentration observed in this study, it resulted in reduction of parasitic load in SL in the CanL group. We showed, *in vitro*, that inhibition of miR-148a played an important role in leishmanicidal activity, however, the mechanisms involved should be better explored, like an IFN-γ dosage, because their parasite killing and macrophage function that already know in CanL [31]. Previously, parasite load reduction was also observed with the inhibition of miR21 [22], suggesting that parasitic load control is multifactorial and appears to be regulated by more than one miRNA in *L*. *infantum* infected dogs.

Decreased iNOS, TNF-α, IL-6, and IL-12 levels after miR-148a mimic transfection in SL suggests a negative regulatory role of this miR, which can act directly or indirectly in immune responses to infection by *L*. *infantum*. To elucidate the biological function of downregulated cytokines in CanL, it would be compelling to neutralize TNF-α, IL-6, and IL-12 and observe whether they influence parasitic load.

The decreased parasitic load in SL following the use of the miR-148a inhibitor suggests a critical role for this miR in developing microbicidal activity in CanL. We conclude that miR-148a regulates immune response as demonstrated by reducing iNOS, TNF-α, IL-6, and IL-12 after miR increase. It plays an essential role in the adaptive response, as demonstrated by the reduction in parasitic load following its inhibition.

## Supporting information

S1 Fig Restriction fragment length polymorphism analysis. ITS1-PCR fragments amplified from DNA samples using Hae III. NC: Negative control (water); M: molecular marker (123 bp); La: *Leishmania amazonensis* (IOC / L0575-MHOM / BR / 1967 / PH8); Lb: *Leishmania braziliensis* (IOC / L0566-MHOM / BR / 1975 / M2903); Li: *Leishmania infantum* (IOC / L0575-MHOM / BR / 2002 / LPC-RPV); C1 to C5: control group; CanL 1 to CanL14: CanL group. CanL samples profile were identical to *L*. *infantum*.
(TIF)

S2 Fig Melting curve of qPCR with SYBR Green. qPCR was performed using a commercially available specific primer for the *Canis familiaris* miR-148a and the endogenous reference RNA SNORD96A (miScript, Qiagen) [21, 22]. The SYBR Green system (miScript SYBR Green PCR Kit, Qiagen) was used in a real-time thermal cycler (Mastercycler-Ep realplex-4S, Eppendorf). Amplification conditions consisted of an initial activation step of 95°C for 15 min followed by 40 cycles of 94°C for 15 seconds, 55°C for 30 seconds, and 70°C for 30 seconds (for denaturation, annealing, and extension, respectively).
(TIF)

S3 Fig CanL do not affect expression of MHCII, iNOS/MHCII+ and transcription factor T-bet/TCD3+. Expression of MHCII (A), iNOS/MHCII^+^ (B), and T-bet/CD3^+^ (C) of the control and CanL groups in splenic leukocytes maintained in culture for 48 h at 37°C and 5% CO_2_, without any treatment (Medium). Graphical data presented as mean ± standard deviation (Mann-Whitney test). There is no significant difference.
(TIF)

S4 Fig MicroRNA-148a mimic and inhibitor transfection did not change MHCII, CD80 in MHCII^+^, iNOS in MHCII^+^, CD3 and transcription factors T-bet and Gata3 in TCD3^+^ SL from control group. Median expression of MHCII (A), CD80/MHCII^+^ (B), iNOS/MHCII^+^ (C), CD3 (D), T-bet/CD3^+^ (E), Gata-3/CD3^+^ (F) in SL cultured from control group. Splenic leukocytes maintained in culture for 48h at 37°C and 5% CO_2_, in presence of miR-148a mimic or inhibitor, or scrambled (SCR). Splenic leukocytes were stained with monoclonal antibodies conjugated to fluorochromes and analyzed by flow cytometry. Graphical data presented as mean ± SD. Friedman test with Dunn’s multiple comparison test. Graphical data presented as mean ± SD. Friedman test with Dunn’s multiple comparison test. There is no significant difference.
(TIF)

S5 Fig MicroRNA-148a mimic and inhibitor transfection did not change expression of MHCII, CD80 in MHCII^+^, CD3 and transcription factors T-bet and Gata3 in TCD3^+^ SL from CanL group. Median expression of MHCII (A), CD80/MHCII+ (B), CD3 (C), T-bet/CD3+ (D), Gata3/CD3+ (E) in infected group. Splenic leukocytes maintained in culture for 48h at 37°C and 5% CO_2_, in the presence of miR-148a mimic or inhibitor, and scrambled (SCR). Splenic leukocytes were stained with monoclonal antibodies conjugated to fluorochromes and analyzed by flow cytometry. Graphical data presented as mean ± SD. Friedman test with Dunn’s multiple comparison test.
(TIF)

S6 Fig The concentration of IL-6 and IL-12 in SL supernatant culture without any treatment (Medium) showed no significant difference between the groups. IL-6 (A) and IL-12 (B) concentration were quantified, by capture ELISA, in SL supernatants cultures from control (dogs without *L*. *infantum* infection) and CanL (dogs naturally infected by *L*. *infantum)* groups. Splenic leukocytes were maintained in culture for 48 h at 37°C and 5% CO_2_, without treatment. Graphical data represents mean ± SD. Asterisk indicates a significant difference (Mann-Whitney test, * p < 0.05).
(TIF)

S7 Fig The concentration of TNF-α, IL-6, IL-12, and IL-1β in the SL supernatant culture of control dogs did not change after miR-148a mimic and inhibitor transfection in control group. TNF-α (A), IL-6 (B), IL-12 (C), and IL-1β (D), were quantified in SL supernatants cultures from CanL group (dogs naturally infected by *L*. *infantum*) and transfected with miR-148a mimic or inhibitor, or SCR in the presence of Hiperfect following 48 h in the culture at 37°C and 5% CO_2_. Graphical data are presented as mean ± SD. Asterisk indicates a significant difference (Friedman test with Dunn’s multiple comparison test, * p < 0.05).
(TIF)

S8 Fig MicroRNA-148a mimic and inhibitor transfection did not change concentration of IL-1β in CanL group. Concentration of IL-1β was quantified in SL supernatants cultures from CanL group (dogs naturally infected by *L*. *infantum*) and transfected with miR-148a mimic or inhibitor, or SCR in the presence of Hiperfect following 48 h in the culture at 37°C and 5% CO_2_. Graphical data are presented as mean ± SD. Asterisk indicates a significant difference (Friedman test with Dunn’s multiple comparison test, * p < 0.05).
(TIF)

S1 Table Clinical signs of CanL and control groups, detection of anti-Leishmania antibodies (ELISA and DPP) and *L*. *infantum* DNA (PCR-RFLP). CanL: Canine Leishmaniasis. Control: healthy negative control. OD: optical density. Female (F). Male (M). *ELISA cut-off value: OD 0.270. **PCR-RFLP. Restriction fragment length polymorphism (RFLP) analysis of *Leishmania infantum* ITS1-PCR fragments amplified from DNA samples by using Hae III enzyme. ***DPP: Dual-Path Platform.
(PDF)

S2 Table Biochemical profile of CanL and control groups. CanL: Canine Leishmaniasis. Control: healthy negative control. AP: alkaline phosphatase. ALT: alanine aminotransferase, GGT: gamma glutamyl transferase. *Reference values.
(PDF)

S3 Table Red blood cell parameters of CanL and control groups. CanL: Canine Leishmaniasis. Control: healthy negative control. RBC: red blood cells, MCV: mean corpuscular, MCHC: mean corpuscular hemoglobin concentration volume. *Reference value.
(PDF)

S4 Table White blood cells and platelet counts of CanL and control groups. CanL: Canine Leishmaniasis. Control: healthy negative control. *Reference values.
(PDF)

S5 Table Cycle threshold values of curve. Standard curve parameters: Slope -3.372; Y-Intercept 28.57; Efficiency 0.98; R^2 0.955.
(PDF)

S6 Table Individual cycle threshold values of miR-148a, SNORD96A and miR-148a relative expression (miR-148/SNORD96A) of which dogs. CT = Cycle threshold; CanL = CanL: Canine Leishmaniasis group (infected diseased dogs). CG: Control group (healthy uninfected dogs).
(PDF)
