# Supplementary figures and images for: miR-148a regulation interferes in inflammatory cytokine and parasitic load in canine leishmaniasis
Source: PLoS Negl Trop Dis. 2023 Jan 31;17(1):e0011039. doi: 10.1371/journal.pntd.0011039 (PMC9888699; doi:10.1371/journal.pntd.0011039)

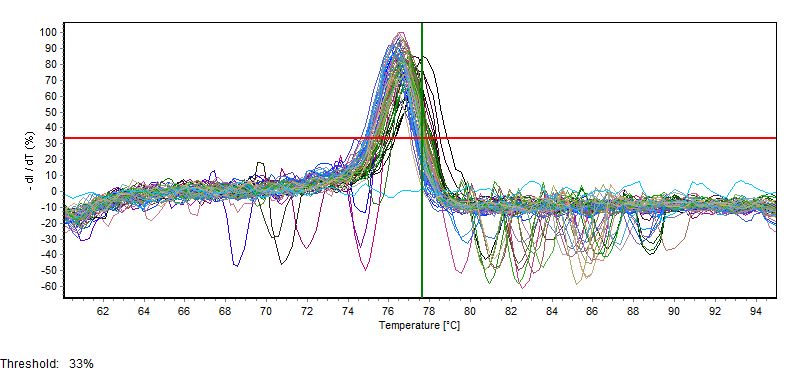

Supplement: S2 Fig — qPCR was performed using a commercially available specific primer for the Canis familiaris miR-148a and the endogenous reference RNA SNORD96A (miScript, Qiagen) [21, 22]. The SYBR Green system (miScript SYBR Green PCR Kit, Qiagen) was used in a real-time thermal cycler (Mastercycler-Ep realplex-4S, Eppendorf). Amplification conditions consisted of an initial activation step of 95°C for 15 min followed by 40 cycles of 94°C for 15 seconds, 55°C for 30 seconds, and 70°C for 30 seconds (for denaturation, annealing, and extension, respectively). (TIF) [file pntd.0011039.s002.tif]
