# Supplementary material for: miR-148a regulation interferes in inflammatory cytokine and parasitic load in canine leishmaniasis
Source: PLoS Negl Trop Dis. 2023 Jan 31;17(1):e0011039. doi: 10.1371/journal.pntd.0011039 (PMC9888699; doi:10.1371/journal.pntd.0011039)
Supplement: S1 Table — CanL: Canine Leishmaniasis. Control: healthy negative control. OD: optical density. Female (F). Male (M). *ELISA cut-off value: OD 0.270. **PCR-RFLP. Restriction fragment length polymorphism (RFLP) analysis of Leishmania infantum ITS1-PCR fragments amplified from DNA samples by using Hae III enzyme. ***DPP: Dual-Path Platform. (PDF) [file pntd.0011039.s009.pdf]

S1 Table. Clinical signs of CanL and control groups, detection of anti-*Leishmania* antibodies (ELISA and DPP) and *L. infantum* DNA (PCR-RFLP).

Dog	CanL				Control			
	Sex	Clinical findings	ELISA OD values*	PCR**/DPP***	Sex	Clinical findings	ELISA OD values*	PCR**/DPP***
1	F	Onychogryphosis, skin lesions, cachexia, seborrhea	1,1325	Reactive	F	None	0,062	Non-detected
2	F	Onychogryphosis, cachexia, skin lesions, seborrhea, alopecia, periocular lesion	1,027	Reactive	F	None	0,026	Non-detected
3	M	Lymphadenomegaly, onychogryphosis, cachexia and skin lesions	1,060	Reactive	M	None	0,147	Non-detected
4	M	Cachexia, seborrhea and skin lesions	0,920	Reactive	M	None	0,028	Non-detected
5	M	Lymphadenomegaly, cachexia, skin lesions, periocular lesion	0,637	Reactive	F	None	0,071	Non-detected
6	M	Lymphadenomegaly, skin lesions, hepatosplenomegaly	0,473	Reactive	-	-	-	-
7	F	Onychogryphosis, cachexia, alopecia, skin lesions	1373,5	Reactive	-	-	-	-
8	M	Lymphadenomegaly, onychogryphosis, cachexia, alopecia, foot injuries	1207,5	Reactive	-	-	-	-
9	F	Lymphadenomegaly, onychogryphosis, seborrhea, alopecia, periocular lesion, hepatosplenomegaly	1,267	Reactive	-	-	-	-
10	F	Lymphadenomegaly, onychogryphosis, periocular lesion, hepatosplenomegaly	1048	Reactive	-	-	-	-
11	F	Lymphadenomegaly, onychogryphosis, cachexia	0,968	Reactive	-	-	-	-
12	F	Lymphadenomegaly, onychogryphosis, seborrhea	1,049	Reactive	-	-	-	-
13	M	Lymphadenomegaly, cachexia, skin lesions and muzzle, hepatosplenomegaly	0,705	Reactive	-	-	-	-
14	M	Onychogryphosis, Cachexia, Seborrhea, Snout Lesions, Periocular Lesions	0,552	Reactive	-	-	-	-

CanL: Canine Leishmaniasis. Control: healthy negative control. OD: optical density. Female (F). Male (M). *ELISA cut-off value: OD 0.270. **PCR-RFLP. Restriction fragment length polymorphism (RFLP) analysis of *Leishmania infantum* ITS1-PCR fragments amplified from DNA samples by using Hae III enzyme. CanL samples profile were identical to *L. infantum.* ***DPP: Dual-Path Platform
