# Supplementary material for: miR-148a regulation interferes in inflammatory cytokine and parasitic load in canine leishmaniasis
Source: PLoS Negl Trop Dis. 2023 Jan 31;17(1):e0011039. doi: 10.1371/journal.pntd.0011039 (PMC9888699; doi:10.1371/journal.pntd.0011039)
Supplement: S2 Table — CanL: Canine Leishmaniasis. Control: healthy negative control. AP: alkaline phosphatase. ALT: alanine aminotransferase, GGT: gamma glutamyl transferase. *Reference values. (PDF) [file pntd.0011039.s010.pdf]

S2 Table. Biochemical profile of CanL and control groups.

Dogs #	Albumin	Globulin	Total protein	AP	Creatinine	Urea	ALT	GGT
	26-33 g/L*	24-44 g/L*	54-71 g/L*	20 - 156 UI/L*	0.5-1.5 mg/dL*	1.67-8.33 mmol/L*	21-102 UI/L*	1.2-6.84 UI/L*
CanL 1	1,5	5,6	7,1	22	0,5	30	37	2,9
CanL 2	1,1	5,4	6,5	57	0,6	22	31	1,9
CanL 3	1,9	6,8	8,7	92	0,7	49	92	1
CanL 4	2,2	4,6	6,8	70	0,5	35	70	1,5
CanL 5	1	7,4	8,4	135	1,5	87	25	1,9
CanL 6	1,2	7,6	8,8	42	1,5	120	30	1,4
CanL 7	1,4	10,8	12,2	76	1	60	19	1,9
CanL 8	2,7	8,8	11,5	51	0,5	28	159	1,7
CanL 9	1,6	9	10,6	17	0,8	63	20	1,9
CanL 10	1,6	6,8	8,4	166	0,6	51	22	3,4
CanL 11	1,9	8,9	10,8	107	0,5	25	25	1,1
CanL 12	1,9	5,1	7	135	0,5	23	27	1,4
CanL 13	1,7	7,3	9	23	0,7	33	22	2,1
CanL 14	1,6	7,2	8,8	26	0,8	16	23	3,2
Control 1	3,3	3,7	7	74	0,9	25	80	2,6
Control 2	2,9	3,8	6,7	148	0,7	30	105	5,9
Control 3	3,4	3,5	6,9	36	1	27	40	2,9
Control 4	2,9	4,1	7	33	1	50	32	1,2
Control 5	2,7	3,9	6,6	85	1,1	21	50	1,9

CanL: Canine Leishmaniasis. Control: healthy negative control. AP: alkaline phosphatase. ALT: alanine aminotransferase, GGT: gamma glutamyl transferase. *Reference values.
