# Supplementary material for: miR-148a regulation interferes in inflammatory cytokine and parasitic load in canine leishmaniasis
Source: PLoS Negl Trop Dis. 2023 Jan 31;17(1):e0011039. doi: 10.1371/journal.pntd.0011039 (PMC9888699; doi:10.1371/journal.pntd.0011039)
Supplement: S3 Table — CanL: Canine Leishmaniasis. Control: healthy negative control. RBC: red blood cells, MCV: mean corpuscular, MCHC: mean corpuscular hemoglobin concentration volume. *Reference value. (PDF) [file pntd.0011039.s011.pdf]

S3 Table. Red blood cell parameters of CanL and control groups.

Dog #	RBC	Hemoglobin	MCV	MCHC
	5.5-8.5 x10^6/µL*	12-18 g/dL*	60-77 fL*	32-36%*
CanL 1	5,5-8,5	7,8	61,73	31,2
CanL 2	4,05	9,1	65,1	36,4
CanL 3	3,84	11	57,62	35,48
CanL 4	5,38	6,7	75,09	30,45
CanL 5	2,93	6,5	61,02	36,11
CanL 6	2,95	9,5	62,79	35,19
CanL 7	4,3	6,2	53,89	34,44
CanL 8	3,34	3,6	52,13	32,73
CanL 9	2,11	10	68,97	35,71
CanL 10	4,06	4,1	57,14	34,17
CanL 11	2,1	6,6	70,92	33
CanL 12	2,82	8,7	65,62	34,8
CanL 13	3,81	7,3	61,05	34,76
CanL 14	3,44	10,2	74,07	74,07
Control 1	4,05	17,8	67,17	33,58
Control 2	7,89	16,9	72,27	34,49
Control 3	6,78	17,8	65,91	34,23
Control 4	7,89	17,3	73,75	34,6
Control 5	6,78	17,1	68,55	33,53

CanL: Canine Leishmaniasis. Control: healthy negative control. RBC: red blood cells, MCV: mean corpuscular, MCHC: mean corpuscular hemoglobin concentration volume. *Reference value.
