# Supplementary material for: miR-148a regulation interferes in inflammatory cytokine and parasitic load in canine leishmaniasis
Source: PLoS Negl Trop Dis. 2023 Jan 31;17(1):e0011039. doi: 10.1371/journal.pntd.0011039 (PMC9888699; doi:10.1371/journal.pntd.0011039)
Supplement: S4 Table — CanL: Canine Leishmaniasis. Control: healthy negative control. *Reference values. (PDF) [file pntd.0011039.s012.pdf]

S4 Table. White blood cells and platelet counts of CanL and control groups.

Dog #	Leukocytes	Neutrophils	Lymphocytes	Monocytes	Eosinophils	Basophils	Platelets
	6-17 x10³/µL*	3,000-11,000/µL*	1,000-4,800/µL*	150- 1,350/µL*	150- 1,250/µL*	Rares/µL*	160-430 x10³/µL
CanL 1	9,9	6831	2475	396	198	0	220
CanL 2	17,9	13783	2506	1253	358	0	400
CanL 3	16,2	11178	3402	1296	324	0	300
CanL 4	7,1	4686	2130	284	0	0	300
CanL 5	9,3	6417	1860	837	186	0	280
CanL 6	7,2	4320	1800	288	72	0	140
CanL 7	8,1	5022	2997	81	0	0	180
CanL 8	9,8	7350	2156	294	0	0	160
CanL 9	8,5	6035	2040	425	0	0	200
CanL 10	3	2100	780	30	90	0	220
CanL 11	12,4	9176	2976	248	0	0	160
CanL 12	14,6	10950	3358	146	146	0	280
CanL 13	8,3	6142	1826	249	83	0	140
CanL 14	6	3.780	1800	180	240	0	280
Control 1	16,2	10692	2754	1496	1250	0	200
Control 2	15,7	10048	2983	628	2041	0	300
Control 3	12,2	6588	4800	610	122	0	320
Control 4	16,2	10854	3726	324	1296	0	220
Control 5	10,3	6077	3605	515	103	0	220

CanL: Canine Leishmaniasis. Control: healthy negative control. *Reference values.
