# Supplementary material for: miR-148a regulation interferes in inflammatory cytokine and parasitic load in canine leishmaniasis
Source: PLoS Negl Trop Dis. 2023 Jan 31;17(1):e0011039. doi: 10.1371/journal.pntd.0011039 (PMC9888699; doi:10.1371/journal.pntd.0011039)
Supplement: S5 Table — Standard curve parameters: Slope -3.372; Y-Intercept 28.57; Efficiency 0.98; R^2 0.955. (PDF) [file pntd.0011039.s013.pdf]

S5 Table. Cycle threshold values of curve.

Name	Ct SYBR
0.9/1	28,49
0.9/2	28,49
0.45/1	30,10
0.45/2	30,10
0.225/1	30,65
0.225/2	30,65
0.1125/1	31,83
0.1125/2	31,83
0.05625/1	32,79
0.05625/2	32,79
0.028125/1	33,65
0.028125/2	33,65
0.0140625/1	34,89
0.0140625/2	34,89

Standard curve parameters: Slope: -3,372; Y-Intercept: 28,57; Efficiency: 0,98; R^2; 0,955
