# Supplementary material for: miR-148a regulation interferes in inflammatory cytokine and parasitic load in canine leishmaniasis
Source: PLoS Negl Trop Dis. 2023 Jan 31;17(1):e0011039. doi: 10.1371/journal.pntd.0011039 (PMC9888699; doi:10.1371/journal.pntd.0011039)
Supplement: S6 Table — CT = Cycle threshold; CanL = CanL: Canine Leishmaniasis group (infected diseased dogs). CG: Control group (healthy uninfected dogs). (PDF) [file pntd.0011039.s014.pdf]

S6 Table. Individual cycle threshold values of miR-148a, SNORD96A and miR-148a relative expression (miR-148/SNORD96A) of which dogs.

	miR-148a		SNORD96A		
Name	CT mean	Amount mean	CT mean	Amount mean	Amount mean miR-148a/SNORD96A
CanL 1	31.5	0.136	30.94	0.0114	11.929820
CanL 2	32.07	0.092	30.31	0.0182	5.054945
CanL 3	31.3	0.155	30.96	0.0113	13.716810
CanL 4	29.68	0.472	30.86	0.012	39.333330
CanL 5	29.86	0.415	29.06	0.0451	9.201774
CanL 6	31.82	0.109	30.93	0.0119	9.159664
CanL 7	35.89	0.006	36.02	0.00029	20.689660
CanL 8	35.11	0.011	31.63	0.0069	6.242639
CanL 9	32.89	0.053	31.36	0.00849	25.657890
CanL 10	32.31	0.078	32.8	0.00304	8.187135
CanL 11	33.24	0.042	32.05	0.00513	16.129030
CanL 12	33.13	0.045	32.86	0.00279	48.181820
CanL 13	32.9	0.053	34.14	0.0011	3.878116
CanL 14	31.5	0.14	29.37	0.0361	29.874210
CG 1	34.97	0.012	29.94	0.0235	0.510638298
CG 2	33	0.048	29.8	0.0297	1.616161616
CG 3	30.99	0.194	26.82	0.233	0.832618026
CG 4	35.7	0.007	34.9	0.000789	8.871989861
CG 5	29.21	0.659	30.02	0.0223	29.55156951

CT= Cycle threshold; CanL = CanL: Canine Leishmaniasis group (infected diseased dogs). CG: Control group (healthy uninfected dogs).
